# Analysis of Nutritional Composition and Flavor Patterns by Variety (*Porphyra dentata* and *Porphyra yezoensis*) in Dried Laver from Jeonnam, Korea

**DOI:** 10.3390/foods14030335

**Published:** 2025-01-21

**Authors:** Bo-Seop Kim, Ju-Hye Im, Young-Seung Yoon, Hyunggyun Kim, Jeong-Yong Cho, Ju-Ri Ham, Yu-Jin Heo, Hae-In Lee

**Affiliations:** 1 Food and Nutrition Department, Sunchon National University, Suncheon-si 57922, Republic of Korea; kbsubi1982@mokpoabo.kr (B.-S.K.); wngpp117@gmail.com (J.-H.I.); heo_dbwls@naver.com (Y.-J.H.); 2 Mokpo Marin Food-Industry Research Center, Mokpo-si 58621, Republic of Korea; moinet@mokpoabo.kr (Y.-S.Y.); zhffkwl@mokpoabo.kr (H.K.); juri1106@mokpoabo.kr (J.-R.H.); 3 Department of Integrative Food, Bioscience and Biotechnology, Chonnam National University, Gwangju 61186, Republic of Korea; jyongcho17@jnu.ac.kr

**Keywords:** *Porphyra dentata*, *Porphyra yesoensis*, nutritional characteristic, mineral, flavor

## Abstract

This study analyzed 192 samples of *Porphyra dentata* (P-dent) and 201 samples of *Porphyra yezoensis* (P-yezo) from Jeonnam in Korea. Principal component analysis (PCA) and correlation analysis were conducted to establish a nutritional component dataset for laver. The analysis revealed that P-dent had higher moisture and weight but lower protein content than P-yezo. Ca, Mg, and Fe levels were higher in P-dent, while P, Na, and Zn levels were higher in P-yezo. Fatty acids composition analysis indicated that P-dent contained higher levels of linoleic acid, while P-yezo exhibited higher levels of oleic acid and eicosapentaenoic acid (EPA). P-yezo had significantly higher levels of chlorophyll and carotenoids compared to P-dent. Conversely, P-dent exhibited higher L* and b* color values, resulting in a brighter, more yellowish appearance. Sensory analysis indicated that P-yezo was more intense in saltiness and umami, whereas P-dent had higher sourness and sweetness. The principal component analysis (PCA) results showed a clear distinction between P-dent and P-yezo, and 184 correlations among factors (nutrients, characteristics, etc.) were identified. These results contribute to a new database for evaluating the quality of Jeonnam laver.

## 1. Introduction

The need for sustainable food resources is expected to continue increasing due to environmental impacts [1]. Edible foods produced from the ocean are estimated to play a potential role in meeting global food demand [2]. Among them, seaweed and marine macroalgae are widely recognized as sustainable sources of nutrition, offering a variety of physiological health benefits such as antioxidant activities and anti-obesity support [3,4]. Thus, these marine sources are often described as the “food of the future” [5].

Among seaweeds, laver, a type of red algae, is the most consumed and is primarily found and produced in Asian countries such as Korea, Japan, and China [6]. Laver is a rich source of amino acids, including betaine, taurine, aspartic acid, and glutamic acid, and it is a food with significant health benefits due to its very low lipid content [7]. Furthermore, laver provides an abundant supply of essential nutrients, including vitamins, minerals, polysaccharides, and phenolic compounds [8]. It is commonly consumed in the form of dried sheets, as a snack (e.g., gim snack), or as an ingredient in various dishes (e.g., laver as a side dish, roasted laver, laver soup, etc.) [9]. In South Korea, 20 varieties of laver are produced, among which *Porphyra yezoensis* (P-yezo), known for its soft texture, and *Porphyra dentata* (P-dent), which has a firmer texture, are primarily cultivated [10]. The main laver species produced in Korea recently include *Porphyra tenera*, *Porphyra yezoensis*, *Porphyra seriata*, and *Porphyra dentata*, among which *Porphyra dentata* and *Porphyra yezoensis* are primarily distributed along the southwestern coast and have high production volumes [11]. Therefore, *Porphyra dentata* and *Porphyra yezoensis* were selected as the representative species of the Jeonnam region for the study. Laver is known to contain mycosporine-like amino acids (MAAs), such as shinorine and porphyra-334, which are reported to exhibit antioxidant activities and skin-protective effects [12]. In addition, South Korea’s proposed standards for laver products were adopted as part of the Asian standards by the Codex Alimentarius Commission, contributing to the globalization of laver products in July 2017 [13]. In the past, the consumption of laver was limited to Asian countries, but recently, as it has been processed in various forms such as side dishes, snacks, and beverages, it has gained popularity worldwide and is now one of the most popular seaweeds [14]. With the rising popularity of laver, several studies have examined its quality properties based on factors such as variety [15], harvest time [16], production region [17], quality during processing and storage conditions [18], and characteristics affected by seasoning [19]. Furthermore, the nutritional composition and bioactive compound content of laver are significantly influenced by cultivation methods, harvest timing, and marine conditions [20]. Jung et al. reported that, when comparing the nutritional components of laver from Wando and Busan, laver produced in Wando had significantly higher levels of calcium, sodium, and potassium than that produced in Busan [21]. However, research comparing nutritional components based on varietal differences remains limited. Therefore, this study aimed to establish a comprehensive database for Jeonnam laver, incorporating nutritional characteristics, color values, and sensory attributes.

## 2. Materials and Methods

### 2.1. Collection of Laver Samples

*Porphyra dentata* and *Porphyra yezoensis* were collected in November 2021 from the marine areas of six cities in Jeollanam-do, South Korea, namely, Goheung, Shinan, Wando, Jangheung, Jindo, and Haenam. They were then transported to dried laver processing facilities in Jeonnam, where they underwent a drying process. The lavers were dried under the following conditions: a temperature of 45–50 °C for 2 h and a moisture level of 40–45%. A total of 393 samples (192 of *Porphyra dentata* and 201 of *Porphyra yezoensis*) were stored at −80 °C prior to conducting the experiments.

### 2.2. Determination of Moisture, Protein, and Weight Composition

The moisture and protein composition were analyzed according to the methods prescribed by the Association of Official Analytical Chemists (AOAC). The moisture content of the samples was evaluated by measuring the weight loss after 12 h of drying at 105 °C in the oven (WOF-155, DAIHAN, Seoul, Republic of Korea). The protein content was determined using the Kjeldahl method with an automatic nitrogen analyzer (VAPODEST 50S, Gerhardt, Germany). The weight per piece of laver was determined using an electronic scale (Sartorius, Goettingen, Germany). To ensure consistency, all the samples were standardized to the same dimensions, with a length of 210 mm and a width of 190 mm, eliminating any variations in size or shape.

### 2.3. Determination of Minerals

The mineral contents, including zinc (Zn), calcium (Ca), potassium (K), magnesium (Mg), sodium (Na), iron (Fe), were determined using the AOAC method. For the analysis of all the minerals, a microwave digestion method was used for the sample preparation. We used 70% nitric acid (Chemitop Co., Ltd., Jincheon, Republic of Korea) as the digestion reagent, and ultrapure water (18.2 MΩ·cm or higher) was produced using an ultrapure water system (ELGA, High Wycombe, UK). For the sample preparation, 0.1 g of each sample was placed in a microwave vessel, and 10 mL of nitric acid solution was added. The sample was then subjected to microwave digestion using a microwave digestion system (MARS 6, CEM Corporation, Matthews, NC, USA) for 1 h. After digestion, the sample was allowed to cool, and deionized water (ddH_2_O) was added to bring the final volume to 25 mL. The solution was filtered using a 0.45 μm syringe filter (for laboratory use) and used as the final sample. The five minerals (Ca, K, Mg, Na, Fe) were analyzed using Inductively Coupled Plasma Optical Emission Spectroscopy (ICP-OES, Agilent, Santa Clara, CA, USA), while the zinc (Zn) was analyzed using Inductively Coupled Plasma Mass Spectrometry (ICP-MS, Agilent, Santa Clara, CA, USA).

### 2.4. Determination of Fatty Acid Composition

The fatty acid composition was measured by GC (Agilent, 7890A, Santa Clara, CA, USA). The preparation of fatty acid methyl esters was carried out according to the AOAC method. These esters were then separated using a GC equipped with a Zorbax Eclipse XDB C18 column (Agilent Technologies, Waldbronn, Germany) (4.6 mm × 250 mm, 5 µm). The injector and detector temperatures were maintained at 240 °C and 260 °C, respectively. The carrier gas flow rate was set to 0.79 mL/min. Peak identification was performed by comparing the retention times with those of standard fatty acid methyl esters from the “37 Component FAME Mix” and trans FAME MIX k 110 (Supelco, Bellefonte, PA, USA). The relative amounts of each fatty acid were calculated as a percentage of the total fatty acids using “GC Solution” software on Shimadzu gas chromatograph workstations.

### 2.5. Determination of Color Parameter

The color parameters were analyzed using the CIE-L*a*b* system, where L* indicates lightness, a* represents redness and greenness, and b* denotes yellowness and blueness. Visual information on the samples was collected using the IRIS electronic eye (Alpha MOS, Toulouse, France).

### 2.6. Determination of Pigments

Three pigments (chlorophyll a, chlorophyll b, and carotenoids) were examined in the samples. The determination of the pigment content employed HPLC (Agilent, 1260B, Santa Clara, CA, USA) with a Zorbax Eclipse XDB C18 column (Agilent Technologies, Waldbronn, Germany) (4.6 mm × 250 mm, 5 µm) for separation. The pigments were extracted using 95% methanol (Mallinckrodt Baker, Inc., Phillipsburg, NJ, USA) at 4 °C for 24 h in the dark. After extraction, each sample was sonicated and centrifuged, and the resulting supernatant was diluted with deionized water (ddH_2_O). Pigment peaks were identified and quantified by comparing them with standard pigments, which were used to calibrate the concentrations based on the peak areas. The pure standard pigments were obtained from Sigma Chemical and DHI (Hørsholm, Denmark).

### 2.7. Determination of Sensory Evaluation

Sensory evaluation was performed using an electronic tongue (Astree II; Alpha MOS, Toulouse, France) equipped with five potentiometric sensors: sourness, sweetness, saltiness, umami, and bitterness. For the analysis, a sample (5 g) was mixed with 100 mL of purified water and stirred at 60 °C for 1 h, and then assessed using the electronic tongue. Prior to the experiments, conditioning, calibration, and diagnostics were carried out with a standard provided by Alpha MOS. During each measurement, the samples were analyzed for 120 s, with the final sensor values determined as the average of the last 15–20 s of the measurement period.

### 2.8. Determination of Shinorine and Porphyra-334

Laver (0.1 g) was added to 20 mL of distilled water and extracted for 30 min, followed by filtration. The extract was mixed with an amino acid internal standard (Waters Co., Milford, MA, USA), borate buffer, and AccQ·Tag derivatization reagent, and derivatized by heating at 55 °C for 10 min to be used as a sample. Qualitative and quantitative analyses of shinorine and porphyra-334 were conducted by setting conditions using UPLC (ACQUITY UPLC™ system, Waters Co.) and QTOF/MS (Xevo G2-XS QTOF, Waters MS Technologies, Manchester, UK). The content of shinorine and porphyra-334 in the laver were measured using a standard curve with samples provided by Chonnam National University, and the qualifier and quantifier ions were identified using UNIFI software version 26 (Waters Co., Milford, MA, USA). Mol file collection was performed using ChemSpider (https://www.chemspider.com/).

### 2.9. Statistical Analysis

Statistical analyses were conducted using SPSS software version 26 (IBM Co., Chicago, IL, USA). All the experiments were processed in triplicate. The data are presented as means ± standard error (S.E). The differences between the two groups were compared by Student’s *t*-test. The correlation analysis was conducted using Pearson’s correlation coefficient. Principal component analysis (PCA) was processed with R studio software (version 4.3.3).

## 3. Results and Discussion

### 3.1. Content of Moisture, Protein, Weight, and Pigments

Table 1 presents the moisture, protein, weight, and pigment content in the laver. The moisture content of P-dent and P-yezo was 7.99% and 7.28%, respectively, with P-yezo showing a significantly lower value than P-dent. On the other hand, the protein content of P-yezo was considerably higher at 34.87% compared to 34.03% for P-dent. Additionally, the weight of P-dent was significantly greater at 4.00 g, compared to 2.68 g for P-yezo. These proximate compositions could be influenced by various factors such as climate, temperature, geographical differences, species, and drying method [22,23]. In the study by Baek et al., a comparison between P-dent and P-yezo also showed that P-dent had a higher moisture content, consistent with our results [7]. P-dent has larger and rougher particles, whereas P-yezo has smaller particles and a smoother texture [24]. P-dent is believed to have a higher weight due to its larger particle size, which allows it to retain more moisture. According to the standards of the Korea Industrial Standard, high-quality dried nori is evaluated based on a moisture content of 12% or less and a protein content of 30% or more [25]. Both P-dent and P-yezo, produced in the Jeonnam region, have been assessed as high quality.

The pigment content, including chlorophyll and carotenoids, has been reported to vary depending on the laver species [26]. In our results, P-yezo showed significantly higher levels of chlorophyll a, chlorophyll b, and carotenoids at 109.72 µL/g, 8.53 µL/g, and 40.78 µL/g, respectively, compared to P-dent, which had levels of 74.39 µL/g, 7.08 µL/g, and 29.36 µL/g. The pigment components in laver, such as chlorophyll and carotenoids, are known to have antioxidant and anti-cancer effects, removing reactive oxygen species to prevent cell damage and reduce inflammation in the body [27]. It is known that the higher the content of photosynthetic pigments such as chlorophyll, carotenoids, and phycobilins, the darker the color of the dried laver [28]. In our results, a negative correlation was observed between pigment content (chlorophyll a, chlorophyll b, carotenoids) and lightness, with a significance level of *p* < 0.01.

### 3.2. Mineral Composition

**Figure 1:**
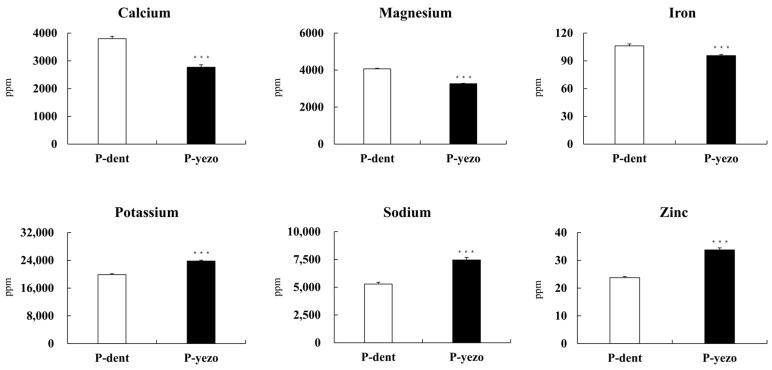
Analysis of mineral content of P-dent and P-yezo. Data values are expressed as means ± S.E. The values are significantly different between groups according to Student’s *t*-test: *** *p* < 0.001 by Student’s *t*-test, P-dent vs. P-yezo. Zinc: Zn, Sodium: Na, Potassium: K, Magnesium: Mg, Calcium: Ca, Iron: Fe, P-dent: *Porphyra dentata* groups, P-yezo: *Porphyra yesoensis* groups.

Laver contains a higher mineral content compared to land plants and animal-based products [29]. Therefore, seaweeds such as laver could serve as a significant source of minerals, as some of these trace elements are either lacking or present in very small amounts in terrestrial vegetables [30]. Thus, we conducted an analysis to measure the mineral composition of the two groups (P-dent and P-yezo). Six elements (Zn, Na, K, Mg, Ca, and Fe) were found in both species of laver (Figure 1). In P-dent, the primary minerals were calcium, magnesium, and iron, while potassium, sodium, and zinc were more prominent in P-yezo. In studies by Kim et al., the analysis of commercial laver’s nutritional components showed that the main minerals in laver were the same as those detected in our results, including Ca, Mg, Na, and P [16]. Seaweed is an important source of plant-based calcium, containing anywhere from 7% to as much as 35%, and laver is also known to have a high calcium content [31]. Significantly, P-dent had 1.4 times higher calcium content (3797 ppm) compared to P-yezo (2770 ppm). Kim et al. also reported that the calcium content of *Porphyra dentata* is higher than that of *Porphyra yezoensis* [20]. Additionally, the magnesium content in P-dent was effectively higher, measuring 4067 ppm, compared to 3266 ppm in P-yezo. The iron content in P-dent, at 106 ppm, was 1.1 times greater than that in P-yezo, which had 96 ppm. On the other hand, P-yezo had 1.2 times higher potassium levels (23,796 ppm) compared to P-dent (19,885 ppm). Furthermore, the sodium and zinc levels in P-yezo were 1.4 times higher, at 7454 ppm and 34 ppm, respectively, compared to P-dent, which had 5288 ppm and 23 ppm. In contrast, Baek et al. reported that sodium, iron, and zinc were abundant in *Porphyra dentata*, while calcium and potassium were more prevalent in *Porphyra yezoensis* [15]. The study by Jung et al. reported that the mineral content of laver can vary significantly even within the same species depending on the region [32]. In particular, laver produced in areas like Wando and Jangheung in Jeollanam-do was found to be rich in minerals, as these regions are half-closed back bays where more minerals and organic matter flow in.

### 3.3. Fatty Acid Composition

Fatty acid composition was analyzed in the P-dent and P-yezo groups (Table 2). Dried laver is known for its high unsaturated fatty acid content, which is more than three times greater than its saturated fatty acid content [33]. Consistent with this, our results revealed that unsaturated fatty acid levels were higher than saturated fatty acids in both groups. The unsaturated fatty acid content was 2.06 g/100 g in P-dent and 2.49 g/100 g in P-yezo, with no significant difference observed between the two groups. Analysis of all the laver samples by group showed that the main saturated fatty acid was palmitic acid. Although P-yezo showed a higher content of palmitic acid (C16:0) than P-dent, the difference was not statistically significant, with palmitic acid being the primary saturated fatty acid in seaweed [34]. A comparative analysis of palmitic acid across six types of seaweed found that laver had the highest content [35]. The main unsaturated fatty acids in P-yezo and P-dent were oleic acid (C18:1n-9), linoleic acid (C18:2n-6), dihomo-γ-linolenic acid (C20:3n-6), and eicosapentaenoic acid (C20:5n-3). Seaweed, including laver, has high levels of n-3 and n-6 PUFAs, which are considered essential fatty acids because they cannot be synthesized by mammals and must be obtained through the food chain [36]. Among them, the essential fatty acid linolenic acid was significantly higher in the P-dent group (0.09 g/100 g), being 1.3 times greater than in the P-yezo group (0.07 g/100 g). The P-yezo group exhibited significantly higher levels of oleic acid, dihomo-γ-linolenic acid, and eicosapentaenoic acid compared to the P-dent group. Kim et al. previously reported that eicosapentaenoic acid (EPA) is the main fatty acid in dried laver, with its content varying depending on the collection region [16]. Similarly, our analysis identified EPA as the most abundant fatty acid in both groups. The amount of EPA in P-yezo was significantly higher at 2.00 g/100 g compared to P-dent at 1.66 g/100 g.

### 3.4. Color Parameters and Sensory Properties

**Figure 2:**
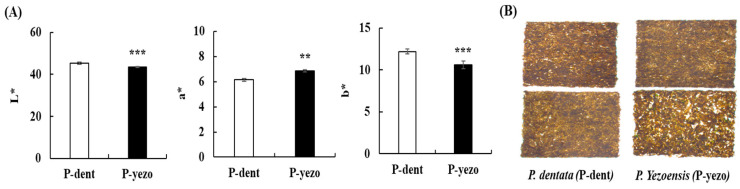
Analysis of color parameters in P-dent and P-yezo. Data values are expressed as means ± S.E. The values are significantly different between groups according to Student’s *t*-test: ** *p* < 0.01 and *** *p* < 0.001 by Student’s *t*-test, P-dent vs. P-yezo. (**A**) Color parameters of P-dent and P-yezo, and (**B**) image of P-dent and P-yezo. L*: lightness, a*: redness/greenness, b*: yellowness/blueness, P-dent: *Porphyra dentata* groups, P-yezo: *Porphyra yesoensis* groups.

Food color plays a crucial role in shaping our perception of taste, and consumers frequently take color into account when selecting food [37]. The color parameters of P-dent and P-yezo are presented in Figure 2A. The L* value, representing brightness, showed significant differences between the two groups, with P-dent exhibiting greater brightness than P-yezo. The b* value, indicating the color yellow, revealed that P-dent exhibited a higher degree of yellowness compared to P-yezo. Conversely, the a* value, which represents the red color, was significantly higher in the P-yezo group compared to the P-dent group. P-dent exhibited higher L* and b* color values, resulting in a brighter, more yellowish appearance. The images of P-dent and P-yezo analyzed using the electronic eye are presented in Figure 2B. The a* value in the color parameter represents the redness of the laver, and it is known that when dried or heated, phycoerythrin decomposes, causing the color to fade [38]. In contrast, the b* value, which represents the yellowness, becomes more intense after the drying or heating process [38]. When comparing the chroma values of P-dent and P-yezo, the a* value was higher in the P-yezo group, while the b* value was observed to be higher in P-dent. These color parameters revealed a potential relationship with certain pigments in the food system [39]. Specifically, chlorophyll and carotenoids are color-related pigments [40]. Carotenoids contribute to red coloration, while chlorophyll a and chlorophyll b appear as bluish-green and yellowish-green, respectively [41,42]. In our results, the higher a* value observed in P-yezo was influenced by its higher carotenoid content. On the other hand, although higher levels of chlorophyll, including chlorophyll a and b, were observed in P-yezo, the b* value indicated greater yellowness in P-dent compared to P-yezo. This is likely due to the longer drying process required for P-dent, which has a higher weight, but further detailed research is needed to confirm this.

**Figure 3:**
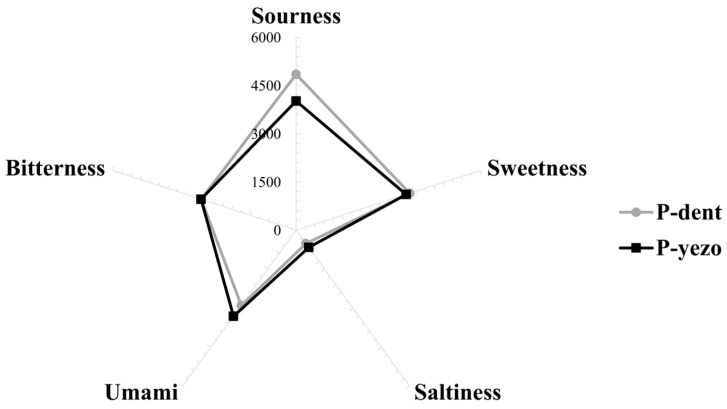
The analysis of sensory property taste (sourness, sweetness, saltiness, umami, and bitterness) in P-dent and P-yezo. P-dent: *Porphyra dentata* groups, P-yezo: *Porphyra yesoensis* groups.

The sensory properties were examined using an electronic tongue sensor to assess sourness, sweetness, saltiness, umami, and bitterness (Figure 3). In the case of saltiness, which shows the main differences between dried lavers, the P-yezo group had a value of 664 and P-dent had a value of 516, indicating a 22% decrease in saltiness in P-dent compared to P-yezo. The umami value of P-yezo was significantly higher, measured at 3315 values compared to P-dent. The umami flavor is enhanced by peptides and their derivatives, which possess nutritional properties, including glutamate [43,44]. Additionally, saltiness is increased by increasing sodium, with NaCl being the main compound in salt [45,46]. In our research, the umami and saltiness values were higher in P-yezo than in P-dent, likely due to the higher protein and sodium content in P-yezo. The umami score demonstrated a positive correlation with the protein (r = 0.154, *p* < 0.01) and sodium (r = 0.149, *p* < 0.01) content and their quantities. In the past, saltiness in dried laver (excluding excessive saltiness) was associated with enhanced umami flavor, increasing consumer preference; however, as the perception of low-sodium products being beneficial to health has grown, this preference has shifted [47]. When processed as snacks rather than side dishes, saltiness might decrease overall consumer preference. The sourness and sweetness of P-dent was higher, with values of 4850 and 3713, compared to P-yezo at values of 4018 and 3593. The bitterness score was not different between the two groups. The functional components of seaweed, such as shinorine and porphyra-334, showed correlations with the sensory properties. Specifically, as the content of shinorine and porphyra-334 increased, the sourness score also increased, indicating a positive correlation (shinorine: r = 0.689, *p* < 0.01; porphyra-334: r = 0.520, *p* < 0.01). On the other hand, they showed negative correlations with bitterness (shinorine: r = −0.744, *p* < 0.01; porphyra-334: r = −0.583, *p* < 0.01) and sweetness (shinorine: r = −0.628, *p* < 0.01; porphyra-334: r = −0.490, *p* < 0.01). This study identified the sensory characteristics of each laver variety; however, a more in-depth analysis of the direct correlation with the contained nutrients is needed.

### 3.5. Principal Component Analysis (PCA) and Correlation Anaylsis

**Figure 4:**
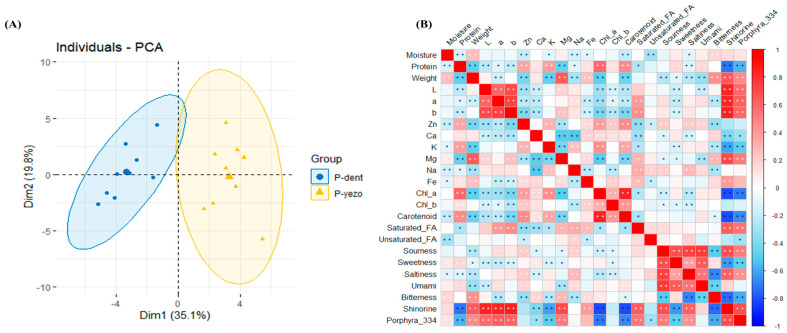
The comparative analysis in P-dent and P-yezo. (**A**) Principal component analysis of P-dent and P-yezo, and (**B**) correlation analysis of P-dent and P-yezo. The color intensity of (**B**) indicates the degree of correlation. Red represents a positive correlation, whereas blue represents a negative correlation. * *p* < 0.05, ** *p* < 0.01. P-dent: *Porphyra dentata* groups, P-yezo: *Porphyra yesoensis* groups.

PCA was performed to investigate the variance of the two groups, P-dent and P-yezo, across different laver characteristics. PCA is commonly utilized to analyze data tables in which observations are defined by multiple dependent variables that often exhibit interrelationships [48]. The PCA of the overall big data measured in this study showed that P-dent and P-yezo accounted for 35.1% and 19.8% of the variance, respectively, demonstrating that the variance between the laver species is distinguishable (Figure 4A). The ellipses in the PCA plots indicate that these variables are significant contributors to group differentiation. Correlation analysis is described as the degree of association between two variables [49].

The relationships between all the measured data were evaluated using comprehensive datasets of the two groups, as shown in Figure 4B, and presented in a heatmap. A total of 184 significant correlations were identified among these variables. Notably, the results revealed a significantly positive correlation between the protein content and the minerals zinc, calcium, potassium, and iron, as well as pigment levels and sensory attributes such as sourness and umami. In contrast, negative correlations were observed between the protein content and weight, color values, the mineral levels of magnesium and sodium, shinorine, and porphyra-334. Another notable finding was the negative correlation between the color values and pigment levels. Shinorine and porphyra-334 were negatively correlated with the color values (chlorophyll a and chlorophyll b), sweetness, and bitterness, whereas they were positively associated with sourness and saltiness. These findings highlight the intricate interconnections between these variables, shedding light on the multifaceted composition of laver and providing valuable insights into its functional and nutritional attributes.

### 3.6. Content of Shinorine and Porphyra-334

**Figure 5:**
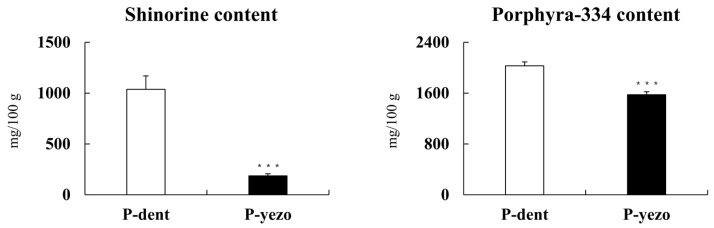
Analysis of shinorine and porphyra-334 content in P-dent and P-yezo. Data values are expressed as means ± S.E. The values are significantly different between groups according to Student’s *t*-test: *** *p* < 0.001 by Student’s *t*-test, P-dent vs. P-yezo. P-dent: *Porphyra dentata* groups, P-yezo: *Porphyra yesoensis* groups.

Mycosporine-like amino acids (MAAs), known as secondary metabolites produced by seaweed, include more than 20 different compounds in this class [50]. Recent studies have highlighted that major compounds in dried laver, such as shinorine and porphyra-334, contribute to its potential health benefits [5]. We measured the levels of shinorine and porphyra-334 using HPLC (Figure 5). Shinorine and porphyra-334 compounds extracted from laver have been shown to inhibit adipogenesis in preadipocytes and suppress inflammation-related genes in human skin cells [14,32]. The analysis of the shinorine and porphyra-334 content revealed significant differences between P-dent and P-yezo. P-dent exhibited significantly higher levels of shinorine, with an average content of 1037 mg/100 g, which was 5.6 times greater than the 186 mg/100 g found in P-yezo. Similarly, the porphyra-334 content in P-dent, at 2030 mg/100 g, was 1.3 times significantly higher compared to P-yezo, which measured 1576 mg/100 g. In the study by Choi et al., the compounds shinorine and porphyra-334 extracted from dried laver were found to inhibit lipid formation in preadipocytes [14]. Also, shinorine and porphyra-334 suppress inflammation-related genes in human skin cells [32]. Therefore, shinorine and porphyra-334 are considered functional components found in dried laver, along with porphyran. These results, depicted in Figure 5, highlight the considerable differences in bioactive compounds concentrations between the two species. Such findings underscore the potential value of P-dent as a richer source of these beneficial compounds.

## 4. Conclusions

This study compared the quality characteristics of P-dent and P-yezo laver, revealing distinct differences in their nutritional and functional properties. P-yezo was found to have higher levels of pigments, protein, essential minerals (potassium, sodium, and zinc), and unsaturated fatty acids, including oleic acid, dihomo-γ-linolenic acid, and eicosapentaenoic acid. It also exhibited a more intense redness in color and stronger saltiness and umami flavors. In contrast, P-dent demonstrated higher levels of calcium, magnesium, and iron, as well as linolenic acid, and higher concentrations of shinorine and porphyra-334. P-dent also exhibited a lighter yellow hue and more pronounced sweetness and sourness. PCA and correlation analysis revealed clear separation between the two types, with significant relationships identified among all the characteristic factors. Notably, diverse correlations were observed among factors such as protein, minerals, color values, pigments, shinorine, and porphyra-334. However, there were no differences in the components between the six regions within the Jeonnam area. These findings provide valuable insights into the nutritional benefits and functional properties of Jeonnam laver, contributing a new database for evaluating its quality and enhancing future studies on laver consumption.

## Figures and Tables

**Table 1:** Analysis of moisture, protein, weight, and pigment content in P-dent and P-yezo *.

	P-dent	P-yezo
Moisture (%)	7.99 ± 0.11	7.28 ± 0.12 ***
Protein (%)	34.03 ± 0.21	34.87 ± 0.33 *
Weight (g)	4.00 ± 0.03	2.68 ± 0.02 ***
Chl-a (µL/g)	74.39 ± 1.81	109.72 ± 1.21 ***
Chl-b (µL/g)	7.08 ± 0.21	8.53 ± 0.20 ***
Carotenoids (µL/g)	29.36 ± 0.84	40.78 ± 0.48 ***

* Data values are expressed as means ± S.E. The values are significantly different between groups according to Student’s *t*-test: * *p* < 0.05 and *** *p* < 0.001 by Student’s *t*-test, *p*-dent vs. P-yezo. Chl-a: chlorophyll a, Chl-b: chlorophyll b, P-dent: *Porphyra dentata* groups, P-yezo: *Porphyra yesoensis* groups.

**Table 2:** Analysis of fatty acid composition of P-dent and P-yezo *.

(g/100 g)	P-dent	P-yezo
C16:0	0.89 ± 0.01	0.91 ± 0.01
C18:0	0.03 ± 0.00	0.03 ± 0.00 *
C23:0	0.16 ± 0.00	0.16 ± 0.00
C24:0	0.01 ± 0.00	0.01 ± 0.00 ***
C20:1	0.09 ± 0.00	0.15 ± 0.00 ***
C20:2	0.03 ± 0.00	0.04 ± 0.00 ***
C18:1n-9	0.08 ± 0.00	0.09 ± 0.00 ***
C18:2n-6	0.09 ± 0.00	0.07 ± 0.00 ***
C22:1n-9	0.02 ± 0.00	0.02 ± 0.00 ***
C20:4n-6	0.00 ± 0.00	0.00 ± 0.00 *
C20:3n-6	0.06 ± 0.00	0.09 ± 0.00 ***
C22:2n-6	0.02 ± 0.00	0.03 ± 0.00 ***
C20:5n-3	1.66 ± 0.02	2.00 ± 0.02 ***
Saturated fatty acid	1.10 ± 0.06	1.11 ± 0.06
Unsaturated fatty acid	2.06 ± 0.06	2.49 ± 0.07

* Data values are expressed as means ± S.E. The values are significantly different between groups according to Student’s *t*-test: * *p* < 0.05 and *** *p* < 0.001 by Student’s *t*-test, P-dent vs. P-yezo. C16:0: Palmitic acid, C18:0: Stearic acid, C18:1n-9: Oleic acid, C18:2n-6: linoleic acid, C20:1: Eicosenic acid, C20:2: Eicosadienoic acid, C20:3n-6: dihomo-γ-linolenic acid, C22:1n-9: Docosaenoic acid, C20:4n-6: Arachidonic acid, C23:0: Tricosanoic acid, C22:2: Docosadienoic acid, C24:0: Lignoceric acid, C20:5n-3: Eicosapentaenoic acid, P-dent: *Porphyra dentata* groups, P-yezo: *Porphyra yesoensis* groups.

## Data Availability

The data presented in this study are available on request from the corresponding author.
